# Light means power: harnessing light spectrum and UV-B to enhance photosynthesis and rutin levels in microtomato plants

**DOI:** 10.3389/fpls.2023.1261174

**Published:** 2023-09-04

**Authors:** Iury Henrique Almeida Lima, Arthur Almeida Rodrigues, Erika Crispim Resende, Fábia Barbosa da Silva, Fernanda dos Santos Farnese, Lucas de Jesus Silva, Márcio Rosa, Mateus Neri Oliveira Reis, Layara Alexandre Bessa, Thales Caetano de Oliveira, Ana Helena Januário, Fabiano Guimarães Silva

**Affiliations:** ^1^ Laboratory of Advanced Studies in Vertical Agriculture, Goiano Federal Institute of Education, Science and Technology, Rio Verde, Brazil; ^2^ Department of Biomolecules, Goiano Federal Institute of Education, Science and Technology, Iporá, Brazil; ^3^ Laboratory of Plant Physiology, Goiano Federal Institute of Education, Science and Technology, Rio Verde, Brazil; ^4^ PostGraduate Program in Plant Production, University of Rio Verde, Rio Verde, Brazil; ^5^ Biodiversity Metabolism and Genetics Laboratory, Goiano Federal Institute of Education, Science and Technology, Rio Verde, Brazil; ^6^ Research Center for Exact and Technological Sciences, Franca University, Franca, Brazil

**Keywords:** flavonoids, LEDs, tomato, rutin, vertical agriculture, *Solanum lycopersicum*

## Abstract

Urban vertical agriculture with lighting system can be an alternative green infrastructure to increase local food production irrespective of environmental and soil conditions. In this system, light quality control can improve the plant physiological performance, well as induce metabolic pathways that contribute to producing phenolic compounds important to human health. Therefore, this study aimed to evaluate the influence of RBW (red, blue and white) and monochromatic (red and blue; R and B, respectively) light associated or not with UV-B on photosynthetic performance and phenolic compound production in microtomato fruits cultivated via vertical agriculture. The experimental design adopted was completely randomized, with six replicates illuminated with 300 µmol·m^−2^·s^−1^ light intensities (RBW, RBW + UV, B, B + UV, R, and R + UV), 12 h photoperiod, and 3.7 W·m^−2^ UV-B irradiation for 1 h daily for the physiological evaluations. Twenty-six days after the installation, gas exchange, chlorophyll a fluorescence and nocturnal breathing were evaluated. Fruits in different ripening stages (green, orange, and red) were collected from microtomato plants grown under with different light qualities, to evaluate the physiological performance. The identification and quantification of the phenolic compound rutin was also performed to investigate their metabolic response. This study identified that plants grown under B + UV had high photosynthetic rates (*A*=11.57 µmol·m^−2^·s^−1^) and the fruits at all maturation stages from plants grown under B and B + UV had high rutin content. Meanwhile, the activation of suppressive mechanisms was necessary in plants grown under R because of the high nocturnal respiration and unregulated quantum yield of the non-photochemical dissipation of the photosystem II. These results highlight the importance of selecting light wavelength for vegetable cultivation to produce fruits with a high content of specialized metabolites that influence color, flavor, and health promotion, which is of special interest to farmers using sustainable cropping systems.

## Introduction

Specialized metabolites, traditionally referred to as secondary metabolites, are compounds present in some natural sources that benefit both survival and reproduction in different environments. These molecules are derived from the primary metabolism, and plants synthesize approximately 200,000 to 1 million such compounds (Garagounis et al., 2021). Phenolic compounds, particularly phenolic acids and flavonoids are a class of compounds of interest due to their high antioxidant capacity that modulates and/or regulates oxidative stress (Ribeiro et al., 2019; Shih and Morgan, 2020). Rutin, a glycoside flavonoid widely distributed in the plant kingdom, is a natural molecule of great interest due to its pharmacological activities and used for treating varicose veins, internal bleeding, and hemorrhoids. It also has antioxidant, anti-inflammatory, antidiabetic, and cardioprotective activities (Semwal et al., 2021).

Elicitors have been a promising strategy to increase bioactive compound production. Light is one of the most studied factors; thus, information is available for several plant species grown under different wavelengths (Liu et al., 2019). Besides being a key factor in regulating growth and development, light also acts as an environmental signal that triggers the production of specific molecules in response to the environment and influences plant development stage (Silva et al., 2020). Wavelength differences are perceived due to the action of several photoreceptors, such as phytochromes (that absorb red and far-red light), cryptochromes, and phototropins (that absorb blue and UV-A light), and UVR8 (that absorbs UV-B light) (Wang et al., 2020). Light also strongly affects the biosynthesis and accumulation of several secondary metabolites in plants, which is related to both increased photosynthesis that generates more carbon skeletons for plant metabolism, including secondary metabolism, and the action of photoreceptors that can perceive photons and trigger signaling pathways, which in turn change gene expression levels. Thus, light condition modulation can stimulate both growth and production of specific compounds of interest (Silva et al., 2020; Thoma et al., 2020). Several studies confirm that photosynthesis can be modulated, and secondary metabolite synthesis is enhanced in species grown under light-emitting diodes (LEDs), particularly when monochromatic blue or red lights are used instead of white light. The individual effects of each wavelength vary depending on the species studied and their growing conditions (Wu et al., 2007; Li and Kubota, 2009; Johkan et al., 2010; Bian et al., 2015; Lobiuc et al., 2017).

Ultraviolet radiation also induces bioactive compound synthesis, since it induces the synthesis of flavonoids and phenolic compounds, which work both as antioxidants and defense molecules for plants (Neugart and Schreiner, 2018). UV-B doses can be manipulated to control secondary metabolite production and plant performance; for example, plants can initially be grown under optimal conditions until they reach a certain degree of development, and then be subjected to UV-B treatment to produce the compounds of interest, thus reducing the morphological impacts of UV-B (Mosadegh et al., 2018). Suzuki et al. (2005) observed a 122% increase in rutin content in UV-B irradiated buckwheat. Moreover, the rutin content also increased in lettuce grown under continuous blue light (Naoya Fukuda et al., 2022). However, high levels of UV-B can be deleterious to plants and are able to influence growth and development, as well as alter genetic, physiological and biochemical aspects, leading to damage to photosynthetic machinery and compromising the photosynthetic rate, triggering photoinhibition and photoprotection responses (Nocchi et al., 2020).

Controlled growing environments mainly circumvent the light-related issues since multiple factors can be controlled. Thus, LEDs can be used because they efficiently convert electrical energy into light energy in addition to having a narrow and specific emission band (Zou et al., 2020). Their use in plant cultivation and the number of studies on the most suitable wavelengths have increased (Thwe et al., 2014; Kim et al., 2015; Ko et al., 2015; Liu et al., 2016; Kim et al., 2018; Liu et al., 2018; Taulavuori et al., 2016; Nam et al., 2018; Taulavuori et al., 2018). Additionally, this protected cultivation environment also circumvents the effects of current climate change scenarios that are expected to worsen in the coming decades, considering that the frequency of natural disasters such as droughts and storms has increased (Fang et al., 2020). It is already well established that blue (425-490 nm) and red (610-700 nm) light are the best spectral qualities for promoting the photosynthetic process of plants (Shafiq et al., 2021). The light environment includes the intensity, quality, and direction of light, with light intensity being the key factor for plant growth (Pan et al., 2020). High light intensities can lead to increased thickness and reduction in leaf area to avoid damage caused by excess light (Matos et al., 2009). Yasar & Uzal (2023) investigated the effect of increased light intensity on tomato plants grown under LED lights with a spectrum similar to the sun and concluded that high intensities can be beneficial, but only if they are within the tolerable limits of the species, since thus the antioxidant system is able to reduce or eliminate the harmful effects of reactive oxygen species (ROS) generated under stress conditions, like that of excess light. For the microtomato, Ke et al. (2022) and Ke et al. (2023) found that 300 μmol.m^-2^.s^-1^ is the appropriate intensity for both the vegetative and reproductive stages, favoring plant growth and increase in fruit biomass. High luminous intensity (700 μmol.m^-2^.s^-1^) led to reduced chlorophyll content and reduced photosynthetic capacity.

Tomato (*Solanum lycopersicum* L.), one of the most consumed vegetables worldwide (>180 million ton being produced yearly), is one of the many crops that can be favored by a controlled environment using LEDs. Its cultivation has become highly effective due to the development of new varieties from breeding programs initiated in the 20th century and the introduction of advanced cultivation technologies (Mata-Nicolás et al., 2020). The main agricultural challenge in the last 20 years has been to produce sufficient nutritious food for a growing population and, at the same time, meet the requirements of sustainable cultivation, thereby reducing environmental impacts (Ronga et al., 2020).

Although rutin is found in many species, few plants are known as major rutin sources (Kianersi et al., 2020). Its concentration increases in UVB-irradiated plants, which reinforces its protective role against UV-B (Chua, 2013). Thus, investigating strategies to increase the production of compounds of interest, such as rutin, is essential to produce nutraceutical foods that will contribute to human health.

In this scenario, we hypothesize that different light wavelengths promote contrasting responses in the production of secondary metabolites such as rutin, the associated use of UV-B light may enhance the secondary metabolite production by increasing plant stress, and using different light wavelengths and UV-B promotes different physiological responses in plants. Thus, this study aimed to evaluate the influence of RBW and monochromatic wavelengths associated with UV-B on the photosynthetic performance and phenolic compound production in microtomato fruits grown in vertical farming.

## Materials and methods

### Plant material and growing conditions

The experiment was conducted at the Plant Biotechnology Laboratory Complex of the Goiano Federal Institute/Rio Verde campus (17°48′10.9″S, 50°54′21.8″W, 755 m altitude). Initially, Micro-Tom (MT) wild type microtomato (MTwt) cultivar seeds were sown in trays containing Bioplant Plus^®^ substrate (BioPlant Agrícola Ltd., Nova Ponte, Brazil) and germinated for 15 days. The seedlings were transplanted to 350 cm³ pots containing the same substrate, supplemented with 0.5 g 4:14:8 NPK, and grown until 39 days in a greenhouse under controlled irradiance, temperature (approximately 25°C), and relative humidity (approximately 60%).

Then, the plants were transferred to an indoor vertical farming system at 50 ± 5% relative humidity and 25 ± 2°C (the room was cooled with air conditioning, to ensure constant temperature and humidity. In addition, the environments with the microtomato plants presented a thermohygrometer for constant monitoring of these variables) in insulated 1.00×0.75 m² vertical boxes with adjustable height and illuminated with LEDs (SG4 – SpectraGrow). Each booth was separated by an expanded polystyrene board (Styrofoam^®^). The plants were exposed to 300 µmol·m^−2^·s^−1^ broad spectrum control LEDs [RBW light (RBW) and RBW + UV-B (RBWUV)], 480 nm blue light (B), blue light + UV-B (BUV), 660 nm red light (R), and red light + UV-B (RUV) for 40 days at constant light intensity, creating approximately 12.96 mol·m^−2^·d^−1^ daily light integral (DLI) and 12 h photoperiod (7:00 AM to 7:00 PM). UV-B light (UV-B Broadband lamp – Philips) was administered daily from 11:00 AM to 12:00 PM (mean intensity: 3.7 W·m^−2^).

The experiment was conducted in an entirely randomized design, with six light qualities and six repetitions. The physiological parameters were evaluated using the leaves 26 days after exposure, considering that most fruits were at their full size. The fruits were collected for rutin content analysis at each growing condition, according to the ripeness level as green, orange, and red.

### Gas exchange and chlorophyll a fluorescence

Gas exchanges were evaluated to record the photosynthesis rate (A, µmol·m^−2^·s^−1^), transpiration rate (E, mmol·m^−2^·s^−1^), stomatal conductance (gs, mol H_2_O·m^−2^·s^−1^), internal CO_2_ concentration (Ci), and nocturnal respiration rate (Rn, µmol CO_2_·m^−2^·s^−1^). These data were used to calculate the water use efficiency (WUE = A/E). Measurements were made using a LI-6800 XT portable gas exchange meter (IRGA, Li-Cor Inc., Nebraska, USA) between 08:00 AM and 11:00 AM on a fully expanded leaf at 25°C, 1000 µmol·m^−2^·s^−1^ artificial photosynthetically active radiation (PAR), approximately 400 μmol·mol^−1^ atmospheric CO_2_ concentration (Ca), and approximately 65% relative humidity.

Chlorophyll a fluorescence was evaluated on the same leaf in which gas exchange was measured using an IRGA. The fluorescence obtained was the effective quantum yield of photochemical energy conversion in photosystem II (FSII) (YII = (Fm′−F)/Fm′). The quantum yields of regulated energy dissipation (YNPQ = (F/Fm′)−(F/Fm)), and unregulated energy dissipation (YNO = F/Fm), were calculated according to the protocols reported by Genty et al. (1989) and Hendrickson et al. (2004), respectively. The ΔF/Fm′ was further used to estimate the apparent electron transport rate (ETR = ΔF/Fm′ *PAR.Leaf_ABS_*.0.5) (Bilger et al., 1995), where PAR is the photon flux density (µmol·m^−2^·s^−1^) incident on the leaf; *Leaf_ABS_* corresponds to the incident light fraction absorbed by the leaves; and 0.5 is the excitation energy fraction distributed to the FSII (Laisk and Loreto, 1996). The apparent electron transport rate to CO_2_ assimilation (ETR/A) rate was calculated to estimate photosynthesis at risk of oxidative stress (Geissler et al., 2015).

### Chloroplast pigment index

Chlorophyll a, b, and total indices were obtained using a CLOROFILOG chlorophyll meter (CFL; Falker 1030, Porto Alegre, Brazil) on a fully expanded leaf, in triplicate. Rutin content analysis

#### Chromatographic conditions

Chromatographic analyses were performed on a Shimadzu^®^ high efficiency liquid chromatograph with SPD-M20A photodiode detector and Zorbax Eclipse Plus C18 analytical column (250×4.6 mm², 5 µm; Agilent^®^) coupled to Zorbax Eclipse Plus C18 pre-column (12.5×4.6 mm², 5 µm; Agilent^®^). The chromatographic conditions were: 10 µL injection volume, 30°C oven temperature, 200–400 nm scanning range, and monitored readings at 254 nm. The mobile phase was eluted at 1.0 mL·min^−1^ flow rate, with phase A comprising 0.1% acetic acid UV/HPLC/spectroscopic grade (Proquimios^®^) and mobile phase B comprising HPLC-grade methanol (J.T. Baker^®^). The elution gradient of mobile phase B increased from 10 to 66% over 32 min, decreased from 66 to 10% in 3 min, and remained isocratic at 10% for 5 min; thus, the total run time was 40 min. The mobile phase with acidified water was filtered using a vacuum mobile filtration system (Restek) with a cellulose filter membrane (47 mm × 0.45 µm, Whatman^®^). The HPLC-grade methanol was filtered through a polytetrafluoroethylene (PTFE) membrane (47 mm × 0.45 µm, Whatman^®^).

#### Analytical curves and sample preparation

The rutin standard (standard bank of the Natural Products Group of the Franca University) was serially diluted to 100, 50, 25, 12.5, 6.25, and 3.125 µg·mL^−1^ using HPLC-grade methanol to construct calibration curves. The resulting solutions were injected in triplicate into the chromatograph. Calibration curves were constructed by plotting the standard chromatographic peak areas versus concentrations in the Microsoft^®^ Excel^®^ software for Microsoft 365 version 2010 for Windows^®^. Linearity was verified by the coefficient of determination (R^2^) and the correlation coefficient (r), which should be above 0.99 according to RDC No. 166/2017 (BRASIL, 2017), with relative standard deviation (RSD) lower than 5%. The resulting equations for each curve were estimated by the least squares method (LSM).

Methanolic extracts were prepared by suspending approximately 0.1 g freeze-dried microtomato in 2 mL HPLC-grade methanol in an ultrasonic bath (Saners Medical, Sonic Clean 2PS) for 30 min. The supernatant was transferred to a 3.0 mL syringe and directly filtered using a syringe containing a Nylon filter (13 mm diameter; 0.45 µm pore diameter; Analítica^®^). Then, 10 μL sample extracts were injected in duplicate in the high-performance liquid chromatograph. The compounds were detected by comparing with the standard retention peak periods and the resulting spectra and quantified through the peak areas and equations obtained from respective analytical curves.

### Statistical analyses

The data obtained were analyzed by the Shapiro–Wilk and Komolgorov–Smirnov tests for normality, and then the homogeneity of variance was verified by the Levene test. These were the necessary assumptions for analysis of variance. These tests showed that the assumptions were not met. Thus, the non-parametric Kruskal–Wallis test was followed by the Dunn (1964) test for multiple comparisons, with methods to control a false discovery rate (FDR) (Benjamini & Hochberg, 1995). The test considered a significance level α equal to 5%. The R statistical software version 4.3.0 (R Core Team, 2023) was used for statistical analyses using the “FSA” (Ogle et al., 2020), “PMCMRplus” (Pohlert, 2018), and “ggplot2” (Wickham, 2011) packages.

## Results

The MT life cycle is accelerated compared to that of conventional tomatoes, since the fruits begin to develop at 39 days and many fruits begin to ripen at 65 days. MT plants were sensitive to R, which was intensified under RUV; thus, evaluating their physiological characteristics were difficult.

### Different wavelength lights with and without UV-B modify the chloroplast pigment index

**Figure 1:**
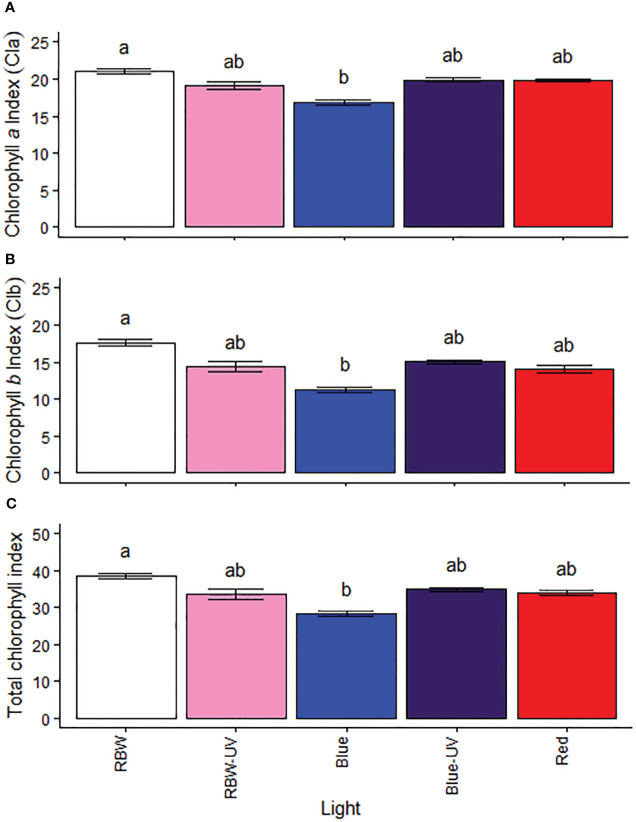
**(A)** Chlorophyll a, **(B)** chlorophyll b, and **(C)** total chlorophyll indexes of microtomato plants after cultivating for 26 days under different light sources with or without UV-B radiation. Means followed by the same letter do not differ by the Dunn’s test at 5% probability.

Microtomato plants grown under RBW had higher chlorophyll a, b, and total chlorophyll index (**Figures 1A–C**), compared to plants grown under B.

### Different wavelength lights with and without UV-B change the light energy used for photosynthesis

**Figure 2:**
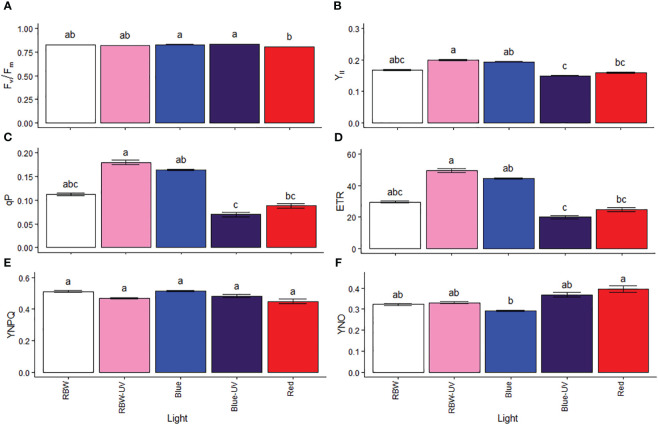
**(A)** Potential quantum yield of photosystem II (Fv/Fm), **(B)** effective quantum yield of photosystem II (YII), **(C)** photochemical quenching (qP), electron transport rate (ETR), quantum yield of regulated energy dissipation (YNPQ), and quantum yield of non-regulated energy dissipation (YNO) in microtomato plants after cultivating for 26 days under different light sources with or without UV-B radiation. Means followed by the same letter do not differ by Dunn’s test at 5% probability.

Chlorophyll a fluorescence was measured to estimate the proportion of light used in photosynthetic processes and its regulatory mechanisms. The maximum FSII quantum yield (Fv/Fm) was low in plants grown under R (**Figure 2A**), indicating sensitivity to light source. However, the plants exposed to BUV and R had lower YII (**Figure 2B**), photochemical quenching (qP, **Figure 2C**), and ETR (**Figure 2D**) than those grown under RBWUV. No difference was observed in quantum yield of regulated energy dissipation (**Figure 2E**). In response to a low YII, qP, and ETR, plants mainly grown under R showed non-regulated energy dissipation (**Figure 2F**), while the lowest values were evident under B.

### Different wavelength lights with and without UV-B affect gas exchange

**Figure 3:**
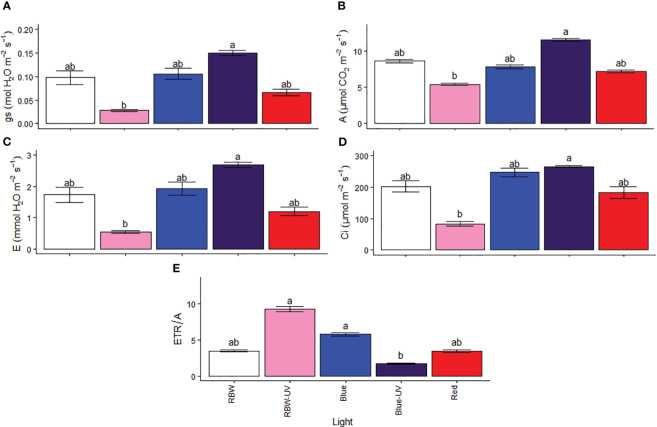
**(A)** Stomatal conductance (gs), **(B)** photosynthetic rate **(A)**, **(C)** transpiration rate **(E)**, **(D)** internal CO_2_ concentration (Ci), and **(E)** electron transport to photosynthetic ratio (ETR/A) for microtomato plants after cultivating for 26 days under different light sources with or without UV-B radiation. Means followed by the same letter do not differ by the Dunn’s test at 5% probability.

Gas exchange variables showed that plants grown under BUV maintained gs (**Figure 3A**), resulting in high A (**Figure 3B**) and E (**Figure 3C**). Plants grown under RBWUV showed lower Ci than plants grown under BUV (**Figure 3D**). In turn, the increased ETR/A (**Figure 3E**) in plants grown under RBWUV may indicate oxidative stress risk.

### Nocturnal respiration rate changes under monochromatic red light

**Figure 4:**
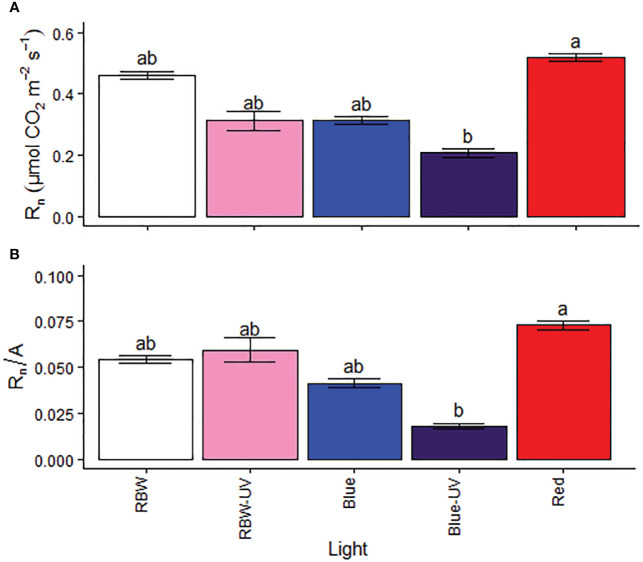
**(A)** Respiration rate (Rn) and **(B)** respiration to photosynthetic ratio (Rn/A) of microtomato plants after cultivating for 26 days under different light sources with or without UV-B radiation. Means followed by the same letter do not differ by the Dunn’s test at 5% probability.

Rn and Rn/A were higher in the plants grown under R, while they were the lowest in the plants grown under BUV (**Figures 4A, B**).

### Principal component analysis of the physiological variables evaluated

**Figure 5:**
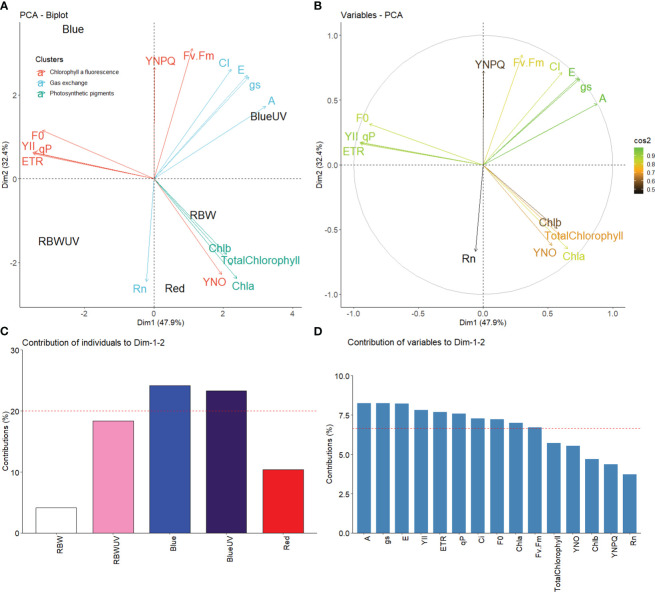
**(A)** Principal component analysis of microtomato chloroplast pigments, chlorophyll a fluorescence, and gas exchange under different light sources with or without UV-B radiation; **(B)** representation quality (cos²) of the variables DIM1 and DM2. The color gradient indicates the quality of variable representations; **(C)** plot of contribution of spectral qualities of light with or without UV-B radiation; **(D)** plot of the contribution of the variables DIM1 and DIM2. The dashed line indicates the expected measured contribution.

The first two dimensions of the principal components (DIM1 and DIM2) together explained 80.3% (DIM1 47.9% and DIM2 32.4%) data variation (**Figures 5A, B**). DIM1 variation was mainly explained by the high influence of BUV on the variables A, gs, and E. W influenced chloroplast pigments (Chla, Chlb, and total chlorophyll). B had the greatest influence on the variables F0, ETR, qP, and YII with DIM2. R directly influenced unregulated energy dissipation and negatively influenced Rn (**Figure 4A**). No variables were influenced by RBWUV. The representation quality of the variable performance (cos², **Figure 5B**) show that A, gs, E, YII, ETR, qP, Ci, F0, and Chla were well-represented in DIM1 and DIM2. B and BUV were the most dominant contributors (**Figure 5C**). A high percentage of contribution (**Figure 5D**) was observed, while Chla, total chlorophyll, YNO, Chlb, YNPQ, and Rn had a low representation (**Figure 5B**).

### Monochromatic blue light and UV-B radiation improve rutin levels in MT fruit

**Figure 6:**
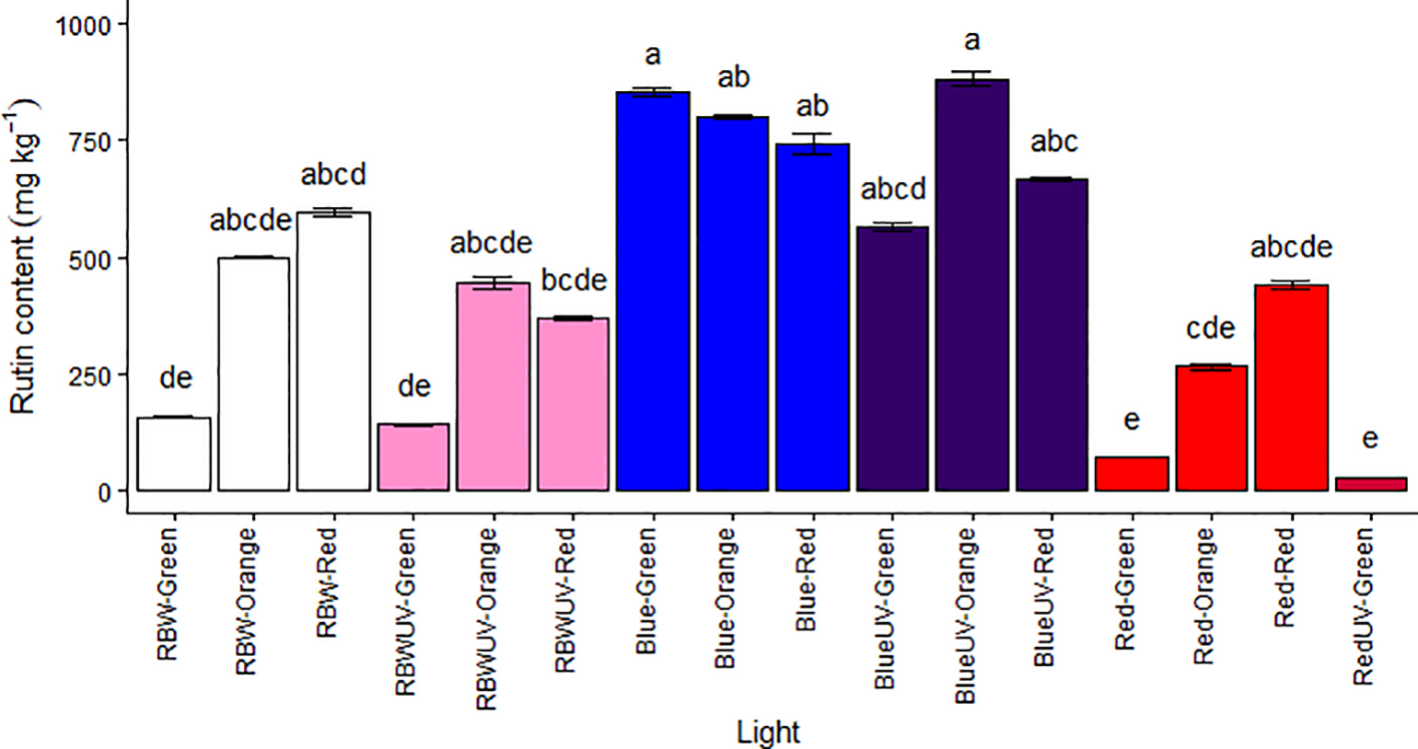
Rutin content (mg·kg^−1^) in the fruits of microtomato plants subjected to six light treatments [RBW light (RBW), RBW + ultraviolet light (RBWUV), blue light (B), blue light + ultraviolet light (BUV), red light (R), and red light + ultraviolet light (RUV)] at different harvesting stages: green tomato (G), orange tomato (O), and red tomato (R). Means followed by the same letter do not differ by the Dunn’s test at 5% probability.

Rutin content was evaluated in green, orange, and red (ripe) MT fruits, except those grown under RUV, due to plant death. In the chromatographic analyzes for the quantification of rutin (RT = 26.9 min e λ_Max_ = 255 e 355 nm), the response of the UV detector at 255 nm was linear from 3,125-100 µg,mL^-1^. The obtained regression equation was y = 21183x – 9983.1 with a correlation coefficient (R2) of 0.9999 (**Supplementary Figures S1–S3**). Higher content of rutin was found in fruits at all maturation stages, both in plants under B and BUV lights, highlighting the green fruits grown under B and orange fruits grown under BUV had the highest rutin concentrations (853.141 ± 37.5 and 882.059 ± 62.2 mg.kg^-1^, respectively) (**Figure 6**).

The response surface graphs for rutin content (mg.kg^-1^) as function of collection stages and light treatments combined or not with UV show that blue light (represented in the graph by the number 2) was the quality of light that most contributed to production of rutin in the three stages of maturation (**Figures S4, S5**). Therefore, they corroborate the results shown in figure 6. Moreover, the green fruits grown under R and RUV had the lowest rutin concentrations (68.234 ± 0.352 and 27.357 ± 1.43 mg.kg^-1^, respectively). Furthermore, the green fruits grown under RBW and RBWUV also had low rutin concentrations (**Figure 6** and **Supplementary Figures S4, S5**).

## Discussion

Various studies have focused on the effects of light spectral quality on the physiological and biochemical performance of plants. Improving the production of bioactive compounds, such as rutin, in fruits is a key step toward healthy food production. This study indicated that despite the positive effects of light wavelength and UV-B radiation on rutin concentration in microtomato fruits, the blue light alone and/or associated with UV-B considerably increased its content. This is probably because monochromatic lights affect photosynthetic parameters, morphology, and stomatal opening rates (Liu et al., 2011; XiaoYing et al., 2011; Wang et al., 2016). Lanoue et al. (2017) have reported high E and Ci in tomatoes grown under monochromatic lights, corroborating the results of this study, in which plants grown under BUV had the highest E (**Figure 3**). Photoreceptors play a fundamental role in plant responses to the environment. Cryptochromes are primarily responsible for mediating blue light-induced changes in gene expression, phenolic compound accumulation, and flowering regulation. Phototropins mediate chloroplast movement and phototropism, both responsible for mediating stoma opening and inhibiting hypocotyl growth. In addition, UVR8 mediates UV-B-induced responses, also changing gene expression and favoring the biosynthesis and accumulation of flavonoids such as rutin (Rai et al., 2021). Blue light-stimulated stomatal opening increases the efficiency of photosynthesis, which are associated with the high absorption capacity of this wavelength by photosynthetic pigments (chlorophylls and carotenoids), leading to decreased chlorophyll content for many species and reduced FSII efficiency (Landi et al., 2020), without affecting CO_2_ fixation efficiency, as observed in this study. The results indicate that pigment and FSII efficiency reductions were adjustments made by the plants to maintain their high photosynthetic efficiency due to high stoma regulation. In contrast, the plants grown under RBWUV light had low gs and A and high FSII efficiency, ETR, and ETR/A, demonstrating that many electrons are transported for energy and NADPH production, not being used for CO_2_ fixation. Shadow avoidance syndrome is often observed in plants grown under R and the negative effects attributed to high phytochrome stimulation are observed in plants whose development have been affected by the absence of far-red light (Izzo et al., 2020).

Hoffmann et al. (2015) have reported that high blue light intensity improved the photosynthetic performance of pepper. MT plants grown under BUV showed similar behavior, with higher gs and, consequently, higher A and E than those grown under other wavelengths (**Figure 3**). This behavior may be related to low photosynthetic pigment degradation and increased production of ultraviolet light-absorbing compounds, which are usually expressed in greater amounts in the presence of both blue light and UV-B light (Nascimento et al., 2013; Ouzounis et al., 2014; Hoffmann et al., 2015). In this study, BUV favored both photosynthetic process and rutin accumulation.

Although the photosynthetic pigments observed in this study (chlorophyll a, b, and total chlorophyll; **Figure 4**) were not the highest in the plants grown under B, they probably induced the production of other pigments, such as carotenoids, which are accessory pigments that invariably increase in response to UV-B because they are associated with light absorption in this spectrum (Kurinjimalar et al., 2019). Palma et al. (2021) have reported that the chlorophyll content variation in peppers treated with associated UV-B and white light is similar to those reported in this study, as the plants grown under RBW had the highest chlorophyll content (**Figure 4**). Although contrary results have been reported in several previous studies, they focus on the use of different UV-B dosages, and the different light to UV-B ratio can lead to different results.

Yang et al. (2018) reported that YII decreased and YNO increased in tomato plants grown under blue and purple light with increasing light intensity compared to those grown under white light. Moreover, the authors reported increased non-photochemically dissipated YNPQ in plants grown under blue and purple light, which indicates photoprotection induction. The results obtained in this study showed similar behavior in microtomato plants grown under BUV with reduced YII and increased YNO, suggesting that the photoprotective capacity of plants grown under BUV improved their development and the accumulation of rutin, a photoprotective metabolite.

According to Igamberdiev et al. (2014), respiration and photorespiration are regulated by gene expression, oxidation state changes, metabolites produced by the photosynthetic process, and photorespiration. Phytochromes mediate the expression of these genes, proving that light perception plays a crucial role in these steps. The high respiration rates in plants grown under R reported in this study indicate high energy demand to handle the stress imposed by this wavelength.

Phenolic compounds are influenced by genetic factors, being associated with the ripening process and phenolic compound metabolism (Scalzo et al., 2005; Atkinson et al., 2006; Paula et al., 2015). Several factors, such as pest attacks, weather stress, ultraviolet radiation, cultivation site, agricultural practices, harvest and storage, processing and preparation conditions and methods, analytical variability, can cause the quantitative variation of a specific flavonoid in a plant; moreover, location and cultivar accounts for 25% and 33% variability, respectively (Haytowitz et al., 2013; Verma et al., 2019). This study confirmed and quantified high rutin contents, particularly in plants grown under B and BUV. Several studies reported similar results, with rutin being the main phenolic compound quantified (Blasa et al., 2010; Vallverdú-Queralt et al., 2012; Flores et al., 2021). Durán et al. (2018) reported higher phenolic compound accumulation in tomato peel than those in seeds and pulp. The whole fruit was analyzed in this study.

Rutin is a compound found in asparagus, buckwheat, Fava d’anta fruit, blackberry, rue, apple, passion fruit flower, grape, and citrus fruits such as orange, lime, lemon, grapefruit. Buckwheat is considered the main rutin source, which despite being present in most plants, is rarely found in the edible parts (Patel & Patel, 2019). Polyphenolic compounds are poorly absorbed or metabolized before reaching their sites of action, due to their low water solubility. Its bioavailability is low in the gastrointestinal tract; thus, approximately 500 mg rutin should be consumed daily, distributed twice a day, to provide its beneficial effects (Remanan & Zhu, 2021). The extraction of bioactive compounds such as rutin requires care, and a series of steps, such as efficiency, cost-effectiveness, and molecule integrity, must be considered for its preservation in the plant (Gullón et al., 2017). Thus, producing fruits with high rutin content is an excellent advantage since it provides health benefits without the need to be extracted. Naoya Fukuda et al. (2022) observed increased expression of genes regulated by blue light. Cryptochromes can induce reactive oxygen species (ROS) synthesis to adapt to stressful environments. Under stress conditions, they can produce phenolic compounds through flavonoid activation and phenylpropanoid biosynthesis. This defense mechanism corroborates the results observed in this study, because the plants grown under B and BUV had high rutin content.

The results of this study show that MT cultivation under blue light with or without UV increases the photosynthetic rate and bioactive compound production. Derived from primary metabolism, compounds that are part of a specialized metabolism use the carbon skeletons generated mainly by the photosynthetic process to be produced. Furthermore, light is an effective elicitor for the increased production of these molecules, as demonstrated in this study and corroborated by many others. The results shown here are fundamental to understanding the influence of different light wavelengths on different crops. In addition, producing bioactive compound-rich foods is important to improve the quality of life of the population that will consume these foods. These results provide a theoretical basis to further elucidate the mechanism behind rutin synthesis regulation by blue light and associated UV-B in tomatoes and establish a new generation of technological innovations for enhancing specialized metabolite production in crops. This work opens the opportunity to improve the nutraceutical quality of fruits, working with the manipulation of phytochromes and with plants more responsive to UVR8 receptors. Furthermore, in future studies it would be possible to verify the effects of increased light intensity, as well as to vary the time of exposure to UV-B to obtain new responses, in addition to those observed in this study. Thus, the tolerable limit for the microtomato plant could be identified, without observing deleterious effects on plants.

## Data availability statement

The original contributions presented in the study are included in the article/**Supplementary Material**. Further inquiries can be directed to the corresponding authors.

## Author contributions

IL: Formal Analysis, Investigation, Methodology, Writing – original draft. AA: Investigation, Methodology, Writing – original draft. ER: Investigation, Methodology, Writing – review & editing. FBS: Investigation, Writing – review & editing. FF: Investigation, Writing – review & editing, Formal Analysis. LS: Investigation, Resources, Writing – original draft. MRo: Writing – original draft, Conceptualization, Methodology. MRe: Writing – original draft, Data curation, Formal Analysis. LB: Data curation, Formal Analysis, Writing – original draft. TO: Resources, Writing – original draft, Investigation. AJ: Conceptualization, Supervision, Visualization, Writing – review & editing. FGS: Conceptualization, Supervision, Writing – review & editing, Funding acquisition.
