# Supplementary figures and images for: Light means power: harnessing light spectrum and UV-B to enhance photosynthesis and rutin levels in microtomato plants
Source: Front Plant Sci. 2023 Sep 4;14:1261174. doi: 10.3389/fpls.2023.1261174 (PMC10507176; doi:10.3389/fpls.2023.1261174)

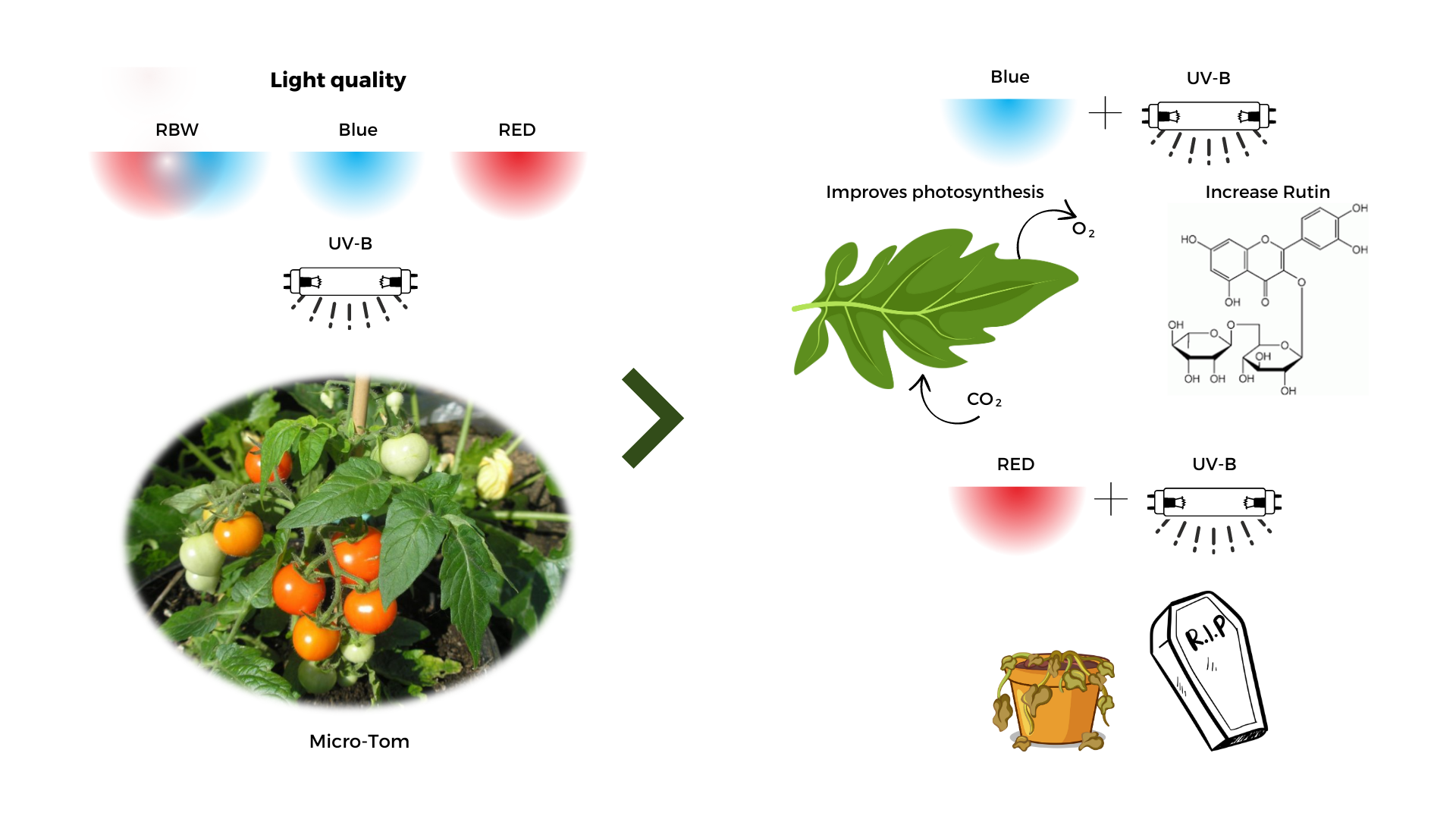

Supplement: Supplementary file 1 [file Image_1.png]
