# Supplementary material for: Light means power: harnessing light spectrum and UV-B to enhance photosynthesis and rutin levels in microtomato plants
Source: Front Plant Sci. 2023 Sep 4;14:1261174. doi: 10.3389/fpls.2023.1261174 (PMC10507176; doi:10.3389/fpls.2023.1261174)
Supplement: Supplementary file 2 [file DataSheet_1.docx]

Supplementary Material

# Supplementary Figures

**Figure S1:** A) Rutin standard calibration curve. Chromatographic conditions: C_18_ (250×4.6 mm², 5 µm); mobile phase: Acidified water (0.1% acetic acid) and methanol, elution programmed in gradient mode, flow rate 1.0 mL·min^−1^, injection 10 µL, oven temperature 30 °C, reading at 254 nm. Solution concentration range: 3.125–100 µg mL^−1^.


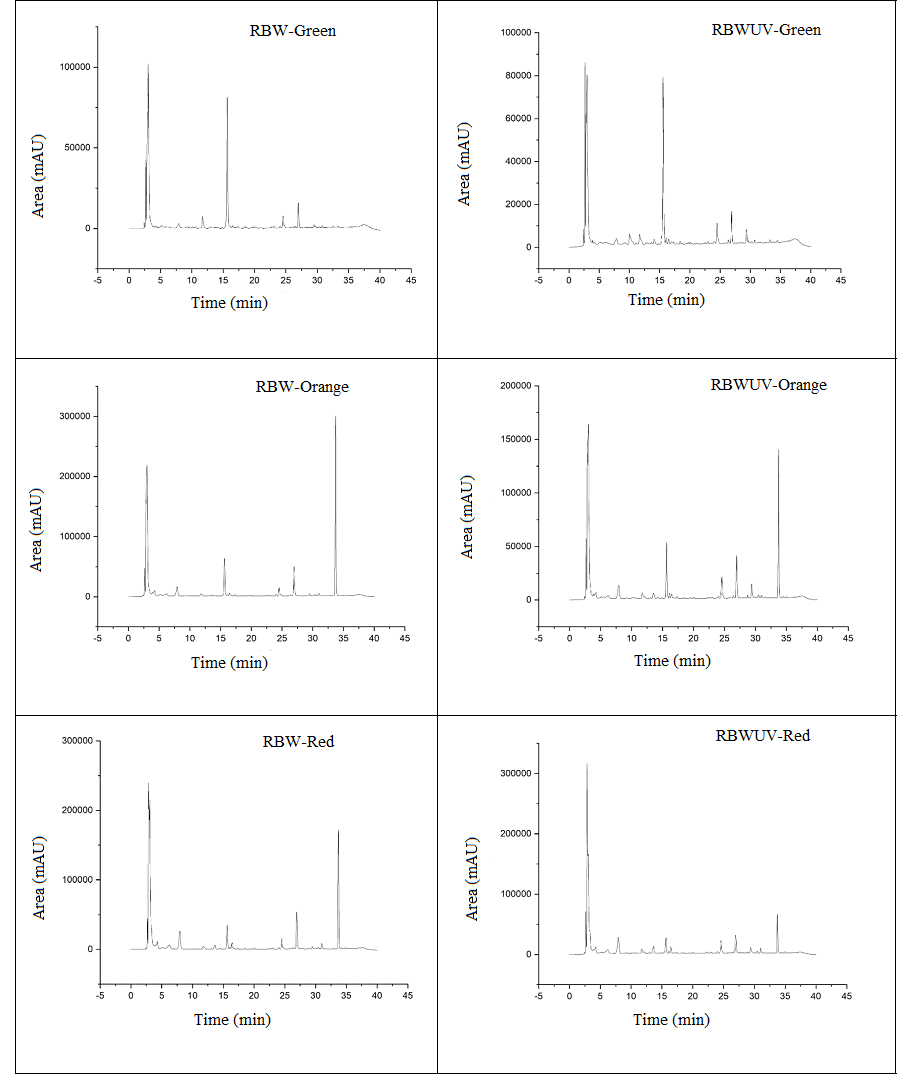


**Figure S2:** Sample chromatograms: RBW-Green; RBWUV-Green; RBW-Orange; RBWUV-Orange; and RBW-Red;and RBWUV-Red samples treated with RBW light and RBW light combined with ultraviolet light. Chromatographic conditions: C_18_ (250×4.6 mm², 5 µm); mobile phase: Acidified water (0.1% acetic acid) and methanol, elution programmed in gradient mode, flow rate 1.0 mL min^−1^, injection 10 µL, oven temperature 30 °C, reading at 254 nm.


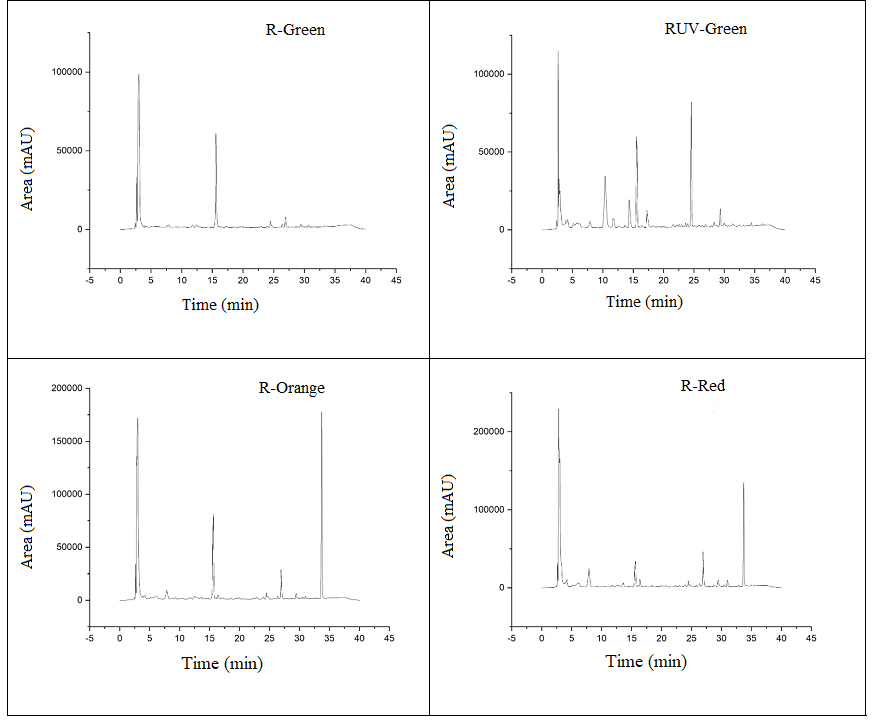


**Figure S3:** Sample chromatograms: R-Green, RUV-Green, R-Orange, and R-Red samples treated with red light and red light combined with ultraviolet light. Chromatographic conditions: C_18_ (250×4.6 mm², 5 µm); mobile phase: Acidified water (0.1% acetic acid) and methanol, elution programmed in gradient mode, flow rate 1.0 mL min^−1^, injection 10 µL, oven temperature 30 °C, reading at 254 nm.


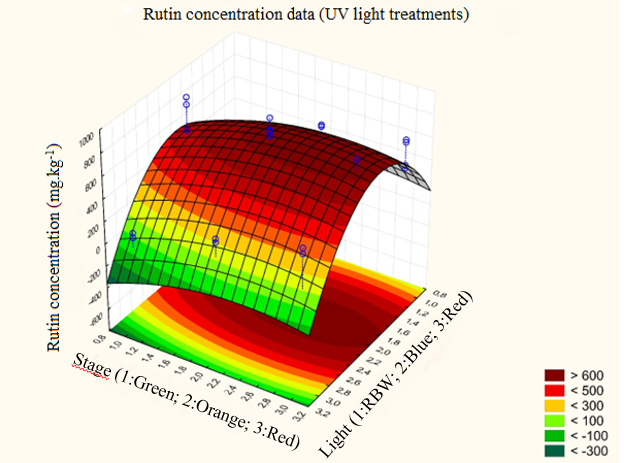


**Figure S4:** Response surface plot for rutin amount (mg kg^-1^) as a function of tomato sample collection stages and light treatments combined with ultraviolet radiation. Data processing performed in the StatSoft software, Inc. (2014). STATISTICA (data analysis software system), version 12. [www.statsoft.com](http://www.statsoft.com)


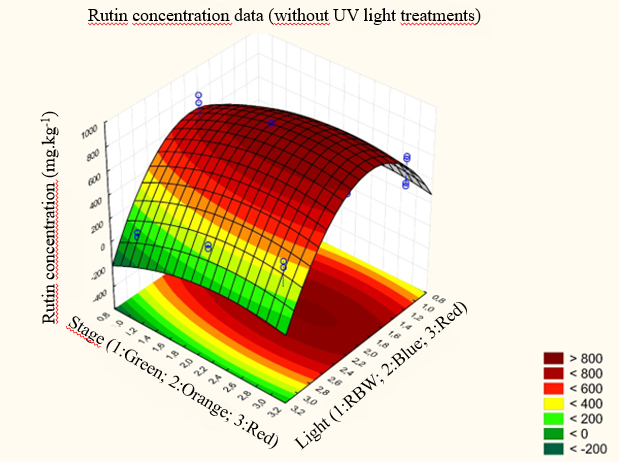


**Figure S5:** Response surface plot for rutin amount (mg kg^-1^) as a function of tomato sample collection stages and light treatments. Data processing performed in the StatSoft software, Inc. (2014). STATISTICA (data analysis software system), version 12. www.statsoft.com.
